# Effects of Fibers on Color and Translucency Changes of Bulk-Fill and Anterior Composites after Accelerated Aging

**DOI:** 10.1155/2018/2908696

**Published:** 2018-01-28

**Authors:** Ali Riza Tuncdemir, Mehmet Esad Güven

**Affiliations:** Dentistry Faculty, Necmettin Erbakan University, Konya, Turkey

## Abstract

The aim of this study was to determine the effects of glass and polyethylene fibers on the color and translucency change of bulk-fill and anterior composites before and after artificial accelerated aging (AAA). Two types of teflon molds were used to fabricate samples which were 13 mm in diameter and, respectively, 2 mm and 4 mm in height. Polyethylene fiber (PF) and glass fiber (GF) were incorporated in the middle of the composite samples. Color and translucency changes of each composite were evaluated before and after AAA with spectrophotometer. ANOVA and Tukey's HSD post hoc statistical analysis were used at a significance level of 0.05. Before AAA (for anterior composites), there were no significant differences in L^*^ and b^*^ parameters among the three groups (*p* > 0.05); there were no significant differences in L^*^ parameter between PF and GF groups or in TP between GF and control groups (*p* > 0.05) (for bulk-fill composites). After AAA, there were no significant differences in L^*^ parameter between GF and control groups, in a^*^ parameter between PF and control groups, in b^*^ parameter among all groups, or in TP parameter between GF and control groups (*p* > 0.05). Fiber reinforcement led to color and TP change in both anterior and bulk-fill resin composites.

## 1. Introduction

Composite resin based materials have been widely used since their introduction to meet the growing demand for esthetic dental treatments [1]. The durability of composite resins is an important factor for their success. Applying fibers, for this reason, to reinforce the composite restorations started in the early 1990s [2]. Using a fiber reinforcement currently has a wide range of dental applications as in implant superstructure, removable partial denture, periodontal splints, and orthodontic retainers and it is an alternative to metal ceramic fix partial dentures [3]. Fibers used in this study were PF and GF. Ribbond-THM is a PF consisting of ultra-high strength braided polyethylene bondable fibers and is not impregnated with resin and must be saturated with an adhesive bonding agent before using. Interlig is a preimpregnated GF.

According to a study, 3 years' survival rates range up to 82.8% for metal ceramic, 88.5% for fiber reinforced, and 72,5% for ceramic resin-bonded prosthesis [4], and also other researches reported successful results and higher patient satisfaction with resin-bonded prosthesis for single tooth replacement than conventional fix partial denture [5].

The Fiber Reinforcement Composites (FRCs) consist of two parts. The fiber part reinforces the composites and provides stiffness; the matrix component (composite) supports the fibers, stabilizes their geometric orientation, and allows workability [3]. Different composite materials can be used for FRCs. It is important to determine which composites are more successful to ensure long-lasting FRC restorations. Color stability of composites affects its clinical longevity and if the color change results in patient dissatisfaction it can be concluded for total or partial replacement [6]. Several intrinsic and extrinsic reasons may cause composites to discolor. Extrinsic factors are related to plaque accumulation, absorption and accumulation of stains and the smoking habits; intrinsic factors are related to the chemical stability of the resin matrix and the matrix/particle interface [7]. Generally, manufacturers recommend that the composites should be placed in 2 mm increments to obtain sufficient light transmission and complete the curing of composite resin but using this incremental technique increases the possibility of air bubble inclusion or moisture contamination between increments of composites and also leads to waste of time [8].

Bulk-fill resin based composites (BRBCs) are innovative class of resin composites and produced to overcome such problems. These materials can be sufficiently light to cure up to 4 mm in a single increment with regard to manufacturers and cause low polymerization shrinkage [9]; the rate of filler content has been reduced to simplify deeper light transmission and particle sizes have been increased to improve the mechanical strength [10]. Recent studies have mostly focused on the depth of cure, degree of monomer conversion, and shrinkage stress, as well as microhardness and cytotoxicity of uncured monomers for BRBCs [11–13] and mechanical properties of FRCs [3]. The differences in filler content and composition are key to the optical feature of resin composites [14]. According to manufacturer both of these composites used in this study have patented innovative initiator system called “Radical Amplified Photopolymerization Technology” (RAP), to offer reduced curing time and excellent stability to ambient light while maintaining the superior esthetic and physical properties. In addition to this feature they include Supra-Nano Spherical filler (200 nm spherical SiO2-ZrO2) with quick curing time, 10 seconds, with a halogen light (≥400 mW/cm^2^) and low polymerization shrinkage different from the other brands.

Translucency is a very important optical property to consider for the color of composite resins. It can be determined with the translucency parameter (TP) and can be described as a color difference in uniform thickness of a material over a white and black background [15]. The TP value is zero when the material is absolutely opaque. The greater the TP value is the higher the actual translucency of a material is. When a material's color has optimal translucency, the restoration will highly resemble the tooth structure and meet the esthetic requirements.

Color stability and translucency are very important for the esthetic restorations but there is no study about color stability of fiber-reinforced bulk filled composites. Therefore, the samples were subjected to artificial accelerated aging (AAA) in order to predict possible alterations on color and translucency change of the composites in a short time in this study.

Recent studies mostly focused on the depth of cure, degree of monomer conversion, and shrinkage stress as well as microhardness and cytotoxicity of uncured monomers for BRBCs [16, 17]. The originality of this study was color stability and translucency is very important for the esthetic restorations but there is no study about effects of fibers on color and translucency changes of bulk-fill and anterior composites before and after AAA. Therefore, the purpose of this study was to determine the effects of fiber incorporation (glass and polyethylene fibers) and AAA on the color and translucency change of anterior composites and bulk-fill, respectively. The null hypothesis is that incorporation of fibers into the composites would not influence these composites' color and translucency.

## 2. Materials and Methods

In this in vitro experimental study, two types of fibers (glass and polyethylene) were incorporated into anterior and bulk-fill composites. Both composites' shades were A2. The characteristics and composition of the materials used in the study are shown in Table 1.

### 2.1. Sample Preparation

Two types of Teflon molds were used to fabricate samples which were 13 mm in diameter and, respectively, 2 mm and 4 mm in height. The spectrophotometer's reservoir diameter was 13 mm, so this width was chosen to allow color measurement. The first layer of the anterior composites was prepared using the shallower (2 mm) mold to enable using incremental polymerization technique and then continued with deeper mold (4 mm) to complete samples. For bulk-fill composites only deeper (4 mm) mold was used to complete samples. Filled mold surface was covered with a Mylar film and the upper and bottom surfaces of the mold were covered by glass slabs before polymerization to produce a smooth surface and finger pressure was applied to extrude excess composite [18]. The samples were polymerized for 20 seconds using a light-emitting diode (LED) curing unit (Elipar S10; 3M ESPE; St. Paul, MN, USA) at a light intensity of 1200 mW/cm^2^ and a wavelength of 430–480 nm (wavelength peak 455 nm). The output of the curing light was tested with a radiometer (1,200 mW/cm^2^). For fibers, both PFs (Ribbond-THM, Ribbond. Seattle, USA) (group PF) and resin impregnated GFs (glass fiber, Angelus, Sao Paulo, Brazil) (group GF) were cut with fiber scissors at 4 mm width, 10 mm length. Ten samples were prepared for each group and totally 60 samples were prepared for this test (*n* = 10).

#### 2.1.1. For Control Groups

No fiber was added to the control groups. 2 mm Teflon mold was inserted into the 4 mm mold and the remaining 2 mm space was filled with composite and polymerized; then the remaining 2 mm composites were added for anterior composites. The residual 4 mm space of the mold was overfilled with bulk-fill composites as monoblock. The composites were cured for 20 s with using the same light curing unit.

#### 2.1.2. For Polyethylene Fiber (PF) Groups

**Figure 1:**
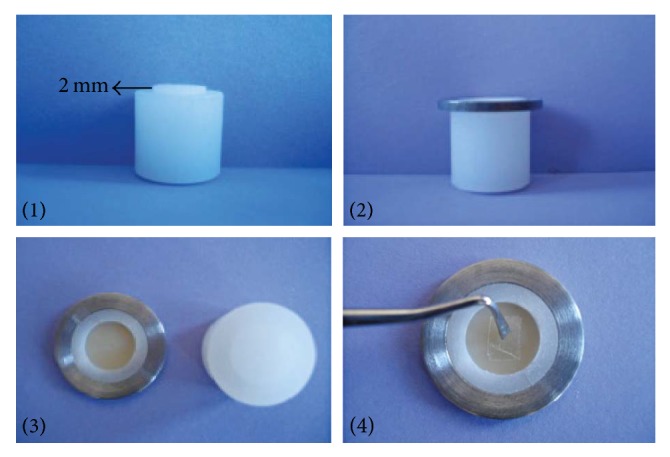
Preparation of samples.

**Figure 2:**
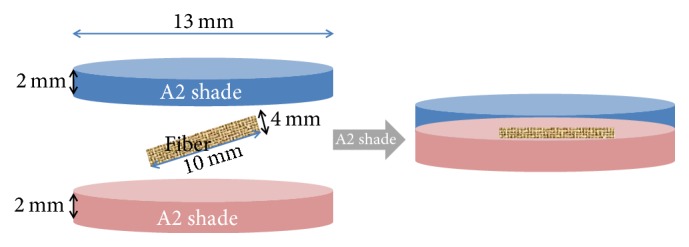
Final view of the samples in the mold.

PF were impregnated with a bonding agent (Clearfil SE Bond) in a small plastic cup. To prepare PF-reinforced composite samples, another custom-made Teflon mold (2 mm height, 13 mm diameter) was inserted into a 4 mm thick mold. After packing a 2 mm thick layer of composite, the custom-made 2 mm Teflon mold was removed. Without curing the bonding agent, PF was placed in the middle of the 2 mm height samples (Figures 1 and 2). After the mold was slightly overfilled with more composite resin, a Mylar strip was put on it and glass slab was clamped on upper surface to throw out excess resin. The composites were cured for 20 s using the same light curing unit.

#### 2.1.3. For Glass Fiber (GF) Groups

Since the GFs were impregnated with resin, they were not subjected to an extra bonding treatment. Composite resin filled and cured applications were performed as defined PF group.

### 2.2. Color and Translucency Measurement

After total 60 composite samples' polymerization, all samples were stored in distilled water at 37°C for 24 hours. Samples were stored in dark boxes until color was measured. Before placing the samples in the spectrophotometer (Lovibond® RT400 Tintometer Colour Measurement Amesbury, UK) it was calibrated according to per manufacturer's instructions and measured solely against white calibration tiles for color evaluation and against white and black calibration tiles for translucency measurements (mean calibration value study device's at D65 condition: white: *L*^*∗*^ = 92,76, *a*^*∗*^ = −1,16, *b*^*∗*^ = 0.70; black: *L*^*∗*^ = 0.38, *a*^*∗*^ = 0.04, *b*^*∗*^ = −0.18). The accurate positioning of samples was enabled by a way of custom jig. Three measurements were conducted at the center of each sample against a white and black background and the mean value was calculated. All samples were evaluated and measured by the same practitioner. Color alterations were determined using the Commission Internationale d'Eclairage *L*^*∗*^*a*^*∗*^*b*^*∗*^ color system (CIE *L*^*∗*^*a*^*∗*^*b*^*∗*^). Color changes were assessed using the following formula [19]:

(1) ΔE=ΔL∗2+Δa∗2+Δb∗21/2,ΔE=L1∗−L2∗2+a1∗−a2∗2+b1∗−b2∗21/2.

This formula was used twice in this study. First, it was used to determine the color differences between control group and fiber-reinforced composite groups at beginning measurement. After that, it was used to determine the color changes of all experimental groups after AAA.

To determine the relationship between the amount of color alteration recorded on a spectrophotometer to the clinical environment, data was converted to the National Bureau of Standards (NBS) system units as follows:

(2) $$\begin{array}{c} NBS\;\;unit=0.92\times \left(\;\Delta E\;\right). \end{array}$$

(See [20].) The defined critical remarks of color change according to the quantified NBS units are given in Table 2 [15]. The TP was calculated as the difference of color coordinate values obtained from the same specimen against black and white background as follows [21]:

(3) TP∗=LB∗−LW∗2+aB∗−aW∗2+bB∗−bW∗21/2,

where the subscript *B* refers to color coordinate values obtained against the black background, and the subscript *W* refers to the values obtained against the white background.

### 2.3. Artificial Accelerated Aging

After baseline color measurement, all specimens were aged for 150 kJ/m^2^ according to accelerated aging conditions previously described [22]. With an accelerated aging chamber (Ci35 Weather-Ometer, Atlas Electric Devices, Chicago, IL, USA), other test parameters included sample surface temperature of maximum 65°C (light) and 38°C (dark) in relative environment humidity of 65%. For rainy condition, surface temperature ranged from 18°C to 38°C. Test cycle was 108 min light plus 65% humidity, 12 min light plus water spray, 108 min dark and 65% humidity, and 12 min dark plus water spray for a total of 150 hours.

### 2.4. Statistical Analysis

Color difference and TP changes of anterior and bulk-fill composites were analyzed with one-way analysis of variance (ANOVA) and Tukey's HSD post hoc test according to fiber type at a significance level of 0.05. To identify the existing differences, post hoc comparisons were performed using the Tukey HSD test and Tamhane's T2 tests. As one-way ANOVA assumes homogeneity of variance, Levene's test was used for homogeneity. The Tukey HSD post hoc test was used when equal variances and specimens' sizes were assumed and the Tamhane's T2 test was used for data where equal variances were not assumed.

## 3. Results

Table 3 shows the means (M) and standard deviations (SD) of *L*^*∗*^, *a*^*∗*^, *b*^*∗*^, Δ*L*^*∗*^, Δ*a*^*∗*^, Δ*b*^*∗*^, Δ*E*, TP, and *p* values of the anterior composites and also the differences between the experimental groups (EG). Before AAA, there were no significant differences in *L*^*∗*^ and *b*^*∗*^ parameters among the three EG (*p* > 0.05). However, the differences in TP and parameters between the groups were statistically significant (*p* < 0.05). After AAA, there were no significant differences in *L*^*∗*^, *a*^*∗*^, and *b*^*∗*^ parameters in all groups (*p* > 0.05). TP chance was statistically significant between GF and control groups. Color change was statistically significant between PF and control groups (*p* < 0.05).

Table 4 shows the M and SD of *L*^*∗*^, *a*^*∗*^, *b*^*∗*^, Δ*L*, Δ*a*, Δ*b*, Δ*E*, and TP values of the bulk-fill composites and the differences among the EG. Before AAA, there were no significant differences in *L*^*∗*^ parameter between PF and glass fiber groups, for a parameter between PF and control groups, for *b* parameter between PF and GF and also GF and control groups, and for TP parameter, between GF and control groups (*p* > 0.05). There were statistically significant differences observed between other groups. After AAA, there were no significant differences in *L*^*∗*^ parameter between GF and control groups, for a parameter between PF and control groups, for *b*^*∗*^ parameter for all groups, and for TP parameter between GF and control groups (*p* > 0.05). There were statistically significant differences observed between other groups (*p* < 0.05).

Table 5 shows the color differences between the fiber-reinforced groups and their respective control groups before AAA. As mentioned in this table, reinforcing with PF and GF to the anterior composites and reinforcing with GF fiber to the bulk-fill composites showed slight color change; on the other hand, reinforcing with PF to the bulk-fill composites showed noticeable color change.

## 4. Discussion

On the basis of the attained data, the null hypothesis tested in the present study was partly rejected. That incorporation of fibers would not change the color of composites' color which was rejected; however, the research hypothesis was accepted with respect to the fact that TP was changed after AAA.

The CIE Lab color system that defines color with using three parameters (*L*^*∗*^, *a*^*∗*^, and *b*^*∗*^) was used in this study because of precise results for color parameters [23]. *L*^*∗*^ defines lightness/darkness ranging from white (+) to black (−), *a*^*∗*^ defines red/green ranging from red (+) to green (−), *b*^*∗*^ defines yellow (+) and blue (−), respectively, and Δ*E* shows color change of the material. It is a method for evaluating color differences based on human perception. Based on the human's eye ability, values 1 < Δ*E* < 3.3 are considered appreciable by skilled operators, but clinically acceptable, Δ*E* > 3.3 values are considered appreciable by nonskilled people and are, hence, not clinically acceptable [1]. In addition, in this study, NBS criteria were used to determine the relationship between the amount of color alteration recorded on a spectrophotometer and the clinical environment.

In this study, aging-dependent color differences are in accordance with some previous findings that accelerated aging resulting in the reduction in *L*^*∗*^ values and increase of *a*^*∗*^ and *b*^*∗*^ values [24, 25]. It was surprising that in these studies aging to specimens was for 300 hours but in this study, aging time is 150 hours (150 kj/m^2^), but the results are the same. Therefore it is unnecessary to age 300 hours to find these results for these materials; on the other hand, different periods and aging methods should be conducted for other materials.

The color stability of composite resins can be related to the material properties, that is, composite matrix, filler composition (size and type volume of charged particles), matrix-filler interface, and degree of polymerization (proportion of remaining unreacted carbon-carbon bonds), shade and to the restorative techniques including the finishing and polishing procedures [26, 27]. The polishing procedures were not investigated in this study; so to achieve the smoothest surface and to standardize the specimens, a Mylar strip was used during light-polymerizing and also to represent clinical situations when matrices were used [28].

Before AAA, incorporation of fibers in anterior composites changed their colors Δ*E* = 0,53 (anterior GF) and Δ*E* = 0,93 (anterior PF) and also with these color differences, they were considered clinically slight (Δ*E* < 1.5); for bulk-fill composites Δ*E* = 1,56 (bulk-fill GF) and Δ*E* = 2,69 (bulk-fill PF) these color differences were noticeable (Δ*E* > 1.5). As mentioned in Results, incorporation of fibers affected anterior composites clinically slightly and bulk-fill composites clinically noticeably. This discrepancy can be more translucency of bulk-fill composites.

According to a study [29], bulk-fill composites had similar color stability to hybrid composite after 40 days of AAA, which is similar to Tiba and others [30], but in this study, after accelerated aging, both of the composites showed clinically noticeable color change and also bulk-fill composites became more colorful (2,92 NBS units) than anterior composites (2,40 NBS units) without fibers. This can arise from more than one factor. Both of the composites contain silica-zirconia and composite filler but in different ratios, as showed in Table 1. Filler weight and percentage of the bulk-fill composites are lower than anterior composites. It may result in surface degradation of the material and absorbing more water, and then greater water sorption provides the composite with lower color stability, due to the increase in free volume of the formed polymer and, as a result, greater space for the water molecules emerges to diffuse into the polymeric network, contributing to degradation of the material [31]. The literature is confusing about the effects of the filler size of the composites on color [22, 31–33]. On the other hand, according to a study, monomer content and surface roughness affect discoloration of composites more than size of the filler particles does [34]. Hydrophilicity of the bulk-fill composite monomers may be more than anterior composites; for example, triethylene glycol dimethacrylate (TEGDMA) absorbs water more than a bisphenol glycidyl methacrylate does (Bis-GMA) and these proportions are also important [35]. In absence of pigments, degree of polymerization (proportion of remaining unreacted carbon-carbon bonds) and greater translucency in bulk-fill composites may be one of the other discoloration factors so discoloration is a multifactorial problem.

After AAA, the FRC and non-FRC groups of both anterior control composites and all of the bulk-fill composites showed clinically noticeable color changes in the range of 1.5–3 NBS units and also anterior PF and GF groups showed clinically appreciable color changes in the range of 3–6 NBC units in this study.

According to this study, the most color changes were observed in anterior PF composites (4.2 NBC units); the least were observed in anterior control group (2,40 NBC units). Thicknesses were chosen nearly the same in specimens (Ribbond-THM = 0,18 mm, glass fiber = 0,2 mm) in order not to affect results. Therefore, differences in their chemical structures and preparation procedures could be the reason why PF-reinforced composites exhibited greater color change than GF-reinforced composites did. PF is hand fabricated and GF is preimpregnated fibers by manufacturers. Improperly saturated fibers (PF) may cause voids in FRC and enhance water sorption, and consequently it became more colorful [18, 36]. On the other hand, this finding could be the result of superior adaptation with minimal space between the composite and the GFs. The refractive index of glass-fibers is different from that of the surrounding composite matrix along with its fillers and opacifiers and favors light penetration through composite, so it can be observed as light-colored compared to control groups. A previous study confirms this result [37].

According to our study, bulk-fill composites' TP were higher than anterior composites and adding to fibers decreased the TP values of specimens. Between the fiber groups, PF groups have less TP values than GF groups. After AAA, TP values of the all groups decreased. According to studies [22, 38] high temperature during accelerated aging could have increased the degree of conversion, leading to a change in the refractive index of the matrix. This, in turn, would make the material less translucent as our study increased scattering as a result. On the other hand, in Korkmaz Ceyhan et al. [39] study, obviously in contrast to our study, AAA did not influence the translucency of composites; it may arise from discrepancies of the shade and composites' content.

There were some limitations in this study. This was an in vitro study and influence of brushing and acids from foods and beverages effects on color stability of samples were not tested. These factors can cause major color change in composites in clinical practice. Examining all these parameters in future studies can lead to providing more precise results as well as in vivo studies.

## 5. Conclusions

According to present findings, the following conclusions were drawn.

(1) Fiber reinforcement led to color and TP change in both anterior and bulk-fill resin composites, but the color changes were below the visual perceptibility threshold (Δ*E* > 3.3).

(2) PFs resulted in more colors and TP change than GFs after incorporation into the composite resins. Therefore, laboratory processed fibers would achieve better optimization of esthetics due to better processing and less voids, they can be preferred in esthetic area. PF can be used in nonesthetic area (palatal and posterior regions).

(3) After AAA, FRC and non-FRC groups of both composite materials became darker (−*L* values), more reddish (+*a*), and more yellowish (+*b*).

(4) After AAA, anterior control and all of the bulk-fill composite groups showed clinically noticeable color changes in the range of 1.5–3 NBS units and also anterior PF and GF groups showed clinically appreciable color changes in the range of 3–6 NBC units.

(5) After AAA, the most color change was observed at anterior PF group (appreciable, 4,57 NBS units); the least was observed in anterior control groups (noticeable, 2,61 NBS units).

(6) After AAA, TP decreased in all groups; before and after AAA, bulk-fill composites were more translucent than anterior composites, and GF fibers were more translucent than PF fibers.

## Figures and Tables

**Table 1:** Characteristics and composition of materials used in this study.

Material	Manufacturer	Composition
Ribbond THM	(Ribbond, Seattle, USA)	(i) Polyethylene fibers

Interlig (Resin Impregnated Glass Fiber)	(Angelus, Sao Paulo, Brazil)	(i) Glass fibers (weight), 60 ± 5% (ii) Impregnated resin (weight) 40 ± 5%: Bis-GMA, diurethane, barium glass, silicon dioxide, catalysts.

Estelite Bulk Fill Flow	(Tokuyama Dental Corporation Tokyo, Japan)	Bis-GMA, Bis-MPEPP, TEGDMA Supra-Nano Spherical filler (200 nm spherical SiO2-ZrO2) Composite filler (including 200 nm spherical SiO2-ZrO2) Filler loading: 70 wt% (56 vol%) Mean particle size: 0.2 *μ*m

Estelite Sigma Quick	(Tokuyama Dental Corporation Tokyo, Japan)	Bisphenol A di(2-hydroxy propoxy) dimethacrylate Bis-MPEPP - 10–30% camphorquinone < 1%, dibutyl hydroxy toluene < 1%, MEQUINOL < 1%, triethylene glycol dimethacrylate 5–10%, Filler loading: 78 wt% (69 vol%) Mean particle size: 0.2 *μ*m

Clearfil SE Bond	(Kuraray Dental, Tokyo Japan)	(i) 10-Methacryloyloxydecyl dihydrogen phosphate (MDP); (ii) 2-hydroxyethyl methacrylate (HEMA); (iii) hydrophilic aliphatic dimethacrylate; (iv) dl-camphorquinone; (v) water Bond (i) 10-methacryloyloxydecyl dihydrogen phosphate (MDP); (ii) bisphenol A diglycidylmethacrylate (Bis-GMA); (iii) 2-hydroxyethyl methacrylate (HEMA); (iv) hydrophobic aliphatic dimethacrylate; (v) dl-camphorquinone; (vi) initiators; (vii) accelerators; (viii) silanated colloidal silica

**Table 2:** National Bureau of Standards (NBS) units and the critical remarks of color change.

Colour difference	NBS unit
Trace	0–0.5
Slight	0.5–1.5
Noticeable	1.5–3.0
Appreciable	3.0–6.0
Much	6.0–12.0
Very much	>12.0

*Note*. This table was extracted from our other study [15].

**Table 3:** Means and standard deviations of *L*, *a*, *b*, Δ*L*, Δ*a*, Δ*b*, TP, and Δ*E* values and differences between groups for anterior composites.

	Anterior PF		Anterior GF		Anterior control		*p*	1-2	2-3	1–3
*L*_1_	64,25	(0,61)	63,93	(0,67)	64,31	(1,19)				
*L*_2_	61,81	(0,67)	62,02	(0,59)	62,62	(0,98)				
Δ*L*	−2,43	(0,96)	−1,90	(0,87)	−1,69	(0,38)				
*a*_1_	3,95	(0,29)	4,24	(0,21)	4,60	(0,15)	*∗*		*∗*	*∗*
*a*_2_	5,27	(0,36)	5,31	(0,20)	5,38	(0,19)				
Δ*a*	1,32	(0,52)	1,07	(0,16)	0,78	(0,14)	*∗*		*∗*	*∗*
*b*_1_	17,76	(0,93)	18,38	(0,73)	18,42	(0,44)				
*b*_2_	21,32	(1,67)	21,30	(0,72)	20,20	(0,62)				
Δ*b*	3,56	(1,61)	2,92	(0,82)	1,78	(0,53)	*∗*			*∗*
TP_1_	6,53	(0,45)	6,75	(0,32)	7,42	(0,43)	*∗*		*∗*	*∗*
TP_2_	5,82	(0,50)	5,71	(0,43)	6,25	(0,50)	*∗*		*∗*	
Δ*E*	4,57	(1,79)	3,68	(1,07)	2,61	(0,50)	*∗*			*∗*

^*∗*^ *p* < 0.05; *n* = 10; anterior PF: incorporation of PF into the anterior composite (Group 1); anterior GF: incorporation of GF into the anterior composite (Group 2); anterior control: anterior composite control groups (Group 3); 1-2: statistical results between Group 1 and Group; 2-3: statistical results between Group 2 and Group 3; 1–3: statistical results between Group 1 and Group 3; *L*_1_, *a*_1_, *b*_1_, and TP_1_: values at baseline measurement; *L*_2_, *a*_2_, *b*_2_, and TP_2_: values after accelerated aging.

**Table 4:** Means and standard deviations of *L*, *a*, *b*, Δ*L*, Δ*a*, Δ*b*, and Δ*E* values and differences between groups for anterior composites.

	Bulk-fill PF		Bulk-fill GF		Bulk-fill control		*p*	4-5	5-6	4–6
*L*_1_	67,12	(0,38)	67,22	(0,42)	68,28	(0,45)	*∗*		*∗*	*∗*
*L*_2_	65,22	(0,33)	65,97	(0,55)	65,92	(0,79)	*∗*	*∗*		*∗*
Δ*L*	−1,91	(0,19)	−1,25	(0,49)	−2,36	(0,75)	*∗*	*∗*	*∗*	
*a*_1_	3,63	(0,13)	3,10	(0,25)	3,66	(0,19)	*∗*	*∗*	*∗*	
*a*_2_	5,37	(0,20)	5,06	(0,22)	5,33	(0,29)	*∗*	*∗*	*∗*	
Δ*a*	1,74	(0,15)	1,97	(0,27)	1,67	(0,19)	*∗*		*∗*	
*b*_1_	19,34	(1,91)	20,77	(1,34)	21,76	(0,59)	*∗*			*∗*
*b*_2_	20,00	(0,82)	20,08	(0,44)	20,71	(0,70)				
Δ*b*	0,66	(1,45)	−0,70	(1,25)	−1,06	(0,54)	*∗*	*∗*		*∗*
TP_1_	13,38	(0,95)	14,51	(0,85)	15,09	(0,53)	*∗*	*∗*		*∗*
TP_2_	11,57	(0,76)	12,70	(0,53)	13,02	(0,80)	*∗*	*∗*		*∗*
DE	2,98	(0,44)	2,71	(0,55)	3,17	(0,48)				

^*∗*^ *p* < 0.05; *n* = 10; bulk-fill PF: incorporation of PF into the bulk-fill composite group (Group 4); bulk-fill GF: incorporation of GF into the bulk-fill composite group (Group 5); bulk-fill control: anterior composite control group (Group 6); 4-5: statistical results between Group 4 and Group 5; 5-6: statistical results between Group 5 and Group 6; 4–6: statistical results between Group 4 and Group 6; *L*_1_, *a*_1_, *b*_1_, and TP_1_: values at baseline measurement; *L*_2_, *a*_2_, *b*_2_, and TP_2_: values after accelerated aging.

**Table 5:** Color differences between control and fiber-reinforced groups in NBS units before aging.

Material	Δ*L*	Δ*a*	Δ*b*	TP	Δ*E*	NBS	Color difference
Anterior PF	−2,43	1,32	3,56	6,52	0,93	0.86	Slight
Anterior GF	−1,9	1,7	2,92	6,74	0,53	0,49	Slight
Bulk-fill PF	−1,91	1,74	0,66	13,37	2,69	2,47	Noticeable
Bulk-fill GF	−1,25	1,97	−0,7	14,51	1,56	1,44	Slight
